# *Helicobacter pylori* in sedentary men is linked to higher heart rate, sympathetic activity, and insulin resistance but not inflammation or oxidative stress

**DOI:** 10.3325/cmj.2016.57.141

**Published:** 2016-04

**Authors:** Andriy Cherkas, Peter Eckl, Françoise Guéraud, Orest Abrahamovych, Victoria Serhiyenko, Ostap Yatskevych, Mykhailo Pliatsko, Sergii Golota

**Affiliations:** 1 Department of Internal Medicine No.1, Danylo Halytskyi Lviv National Medical University, Lviv, Ukraine; 2 Department of Cell Biology, University of Salzburg, Salzburg, Austria; 3 Toxalim, Université de Toulouse, INRA, INP-ENVT, INP-EI-Purpan, Université de Toulouse 3 Paul Sabatier, Toulouse, France; 4 Department of Endocrinology, Danylo Halytskyi Lviv National Medical University, Lviv, Ukraine; 5 Department of Pharmaceutical, Organic and Bioorganic Chemistry, Danylo Halytskyi Lviv National Medical University, Lviv, Ukraine

## Abstract

**Aim:**

To compare anthropometric parameters, body composition, hormonal and inflammatory profiles, oxidative stress indices, and heart rate variability (HRV) in *Heliobacter pylori* (*H.pylori)* positive and negative healthy sedentary participants.

**Methods:**

Among 30 recruited apparently healthy male participants (age between 20 and 40) enrolled in this cross-sectional study, 18 were *H.pylori* negative and 12 were positive (stool antigen test). Participants underwent routine physical examination and body composition determination. The following biochemical parameters were determined in blood: fasting whole blood glucose, glycated hemoglobin, insulin, C-peptide, cortisol, aldosterone, testosterone, thyroid stimulating hormone, C-reactive protein, interleukins 6 and 10, tumor necrosis factor-α, and the urinary level of 1,4-dihydroxynonane mercapturic acid. For HRV evaluation, electrocardiogram in supine position and in orthostatic test was performed.

**Results:**

*H.pylori* contamination was not significantly associated with any changes in anthropometric parameters, body composition, blood pressure, fasting glucose, or glycated hemoglobin levels. No significant difference was found for inflammatory markers as well as 1,4-dihydroxynonane mercapturic acid. *H.pylori*-positive participants, however, had significantly higher heart rate (*P* = 0.009), sympathetic/parasympathetic balance in orthostatic test (*P* = 0.029), fasting insulin level (*P* = 0.037), and HOMA-index (*P* = 0.047).

**Conclusions:**

*H.pylori* contamination is linked to a significantly higher heart rate, sympathetic activation, and increased insulin resistance, while inflammatory and oxidative stress markers remain unaffected in healthy sedentary male subjects.

*Helicobacter pylori* (*H.pylori*) is a widely spread infection causing major gastroenterological diseases like chronic gastritis, peptic ulcer, gastric adenocarcinoma, and mucosa-associated lymphoid tissue lymphoma (1). In addition, several extragastric diseases and pathological conditions have been linked to this microorganism (2). Since *H.pylori* is able to cause local tissue damage through reactive oxygen species generation, local oxidative stress is currently considered as a triggering mechanism for inflammatory processes in the stomach (3). Local oxidative stress associated with low grade inflammation is suggested to be responsible for numerous systemic changes, including metabolic deteriorations like nonalcoholic fatty liver disease, insulin resistance, metabolic syndrome, and diabetes type 2 (2-7). It was previously demonstrated that *H.pylori* induced excessive lipid peroxidation and accumulation of 4-hydroxynonenal (HNE)-histidine adducts in the gastric mucosa of patients with duodenal peptic ulcer (8). Interestingly, shortly after *H.pylori* eradication in four weeks period, manifestations of oxidative stress in these patients persisted (9). The other important finding of these two studies is that in a significant number of the control group participants, consisting of both *H.pylori* positive and negative participants without the history of peptic ulcer disease, mild accumulation of HNE-histidine adducts was observed. It is also possible that, to some extent, HNE production may be a physiological process, and that this molecule may be exploited by mucosal cells as a local regulatory mechanism (10). 1,4-dihydroxynonane mercapturic acid (DHN-MA) in urine, a water soluble metabolite of HNE, is a potential noninvasive biomarker of oxidative stress (11).

Insulin resistance, metabolic syndrome, atherosclerosis, and type 2 diabetes develop gradually and have a long latency period. Until recently, most of the studies have focused on already established pathologic conditions, and relatively little attention has been paid to early stages of these diseases in participants without any clinical manifestations. Early shifts, hence difficult to diagnose, are extremely attractive from the clinical point of view, since their detection allows early interventions, enabling the reversal of the pathological process. Conventional clinical parameters, however, cannot detect these changes. Recently, we have suggested that heart rate variability (HRV) may be one of the methods allowing determination of early metabolic changes (12). Importantly, HRV allows noninvasive examinations, which is important for screening programs. Following this hypothesis we found that in apparently healthy sedentary participants HRV reduction correlated with age, subclinical deteriorations of carbohydrate metabolism, and excessive fat accumulation (13). A significant reduction of HRV reflecting autonomic imbalance was also observed in a number of other diseases and conditions, for example ventricular arrhythmia, heart failure, myocardial infarction, and type 2 diabetes, even without evidence of autonomic neuropathy, liver damage, hypertension, all-cause mortality (14), Marfan syndrome (15), and many other problems. Early interventions may reverse initial metabolic shifts and improve HRV, which was shown in particular in the treatment of *H.pylori*-associated duodenal peptic ulcer (16). Correlations of HRV and metabolic parameters were also shown in athletes, and amaranth seed oil (*Amaranthus cruenthus*, *L*.) was used as a remedy to enhance recovery and to improve HRV (17).

Since *H.pylori* positivity is associated with metabolic syndrome and insulin resistance (5,6), and early metabolic deteriorations cause HRV reduction in sedentary apparently healthy young participants (13), we hypothesize that contamination by this microorganism may cause low grade systemic oxidative stress, inflammation, and HRV reduction in sedentary participants. Therefore the aim of our study was to compare anthropometric parameters, body composition, HRV, carbohydrate metabolism, hormonal and inflammatory profiles, and DHN-MA in *H.pylori* positive and negative healthy sedentary male participants.

## Materials and methods

### Study group characteristics and anthropometric parameters

In our study 30 apparently healthy sedentary male participants aged 20-40, nonsmokers, mainly office and research employees, medical staff, and students were enrolled. The information about the study design, protocol, inclusion and exclusion criteria was distributed among the physicians working at Danylo Halytskyi Lviv National Medical University. Individuals seeking preventive examination without prior significant health issues, consultation regarding healthy lifestyle and nutrition were offered to participate voluntarily in the study. Subject enrollment took place between January and September 2012. Participants were divided into two groups – *H. pylori*-positive (n = 12) and *H. pylori*-negative (n = 18) according to the results of *H. pylori* testing. In order to avoid false-negative results enrolled participants must have not taken any drugs interfering with *H. pylori* (antibiotics, proton pump inhibitors, bismuth salts etc) for at least one month prior to the study. Athletes, men actively training for more than one hour per week, severely obese (body mass index above 35.0 kg/m^2^), laborers, and men suffering from any chronic diseases were not included in the study. The questionnaire used in the study was adapted from “The General Practice Physical Activity Questionnaire” (translated into Ukrainian), developed by the London School of Hygiene and Tropical Medicine as a validated short measure of physical activity available online from *http://www.patient.co.uk/doctor/general-practice-physical-activity-questionnaire-gppaq*. Participants were considered as “sedentary” if their results were classified qualitatively as “inactive” or “moderately inactive” according to the online calculator. Enrolled participants never smoked or quit smoking at least three years prior to the participation in the study, no one was vegetarian, or had any voluntary qualitative or quantitative food restrictions. All participants underwent physical examination including blood pressure (BP) measurement, routine clinical tests, and 6-lead electrocardiography recorded in supine position for accurate heart rate (HR) determination (average from 5 min record). Anthropometric data included height, weight, body mass index (BMI), and age. Height was measured using a standardized stadiometer, weight using an electronic scale (OMRON Corporation, Kyoto, Japan), and BMI was calculated. Standard dynamometer was used to measure arm strength according to conventional technique. Body composition was estimated by the bioelectric impedance method, and the following parameters were determined: fat content (% of body weight), visceral fat (%), and muscle mass (%) and estimated basal metabolism rate on the Body Composition Monitor BF500 (OMRON Corporation). It should be noted that the body composition measurements provide approximate results and are used for rough estimation of body composition. The Ethics Committee of Danylo Halytskyi Lviv National Medical University approved the design and protocol of the study, protocol No. 5, May 17, 2010. Written informed consent form was obtained from all the participants.

### Heart rate variability

HRV parameters were derived from short term ECG records (five-minute intervals) in supine position and during orthostatic test. In the morning hours, not less than 24 hours after the last significant physical exercise, the short-time records of ECG were performed in a quiet dark room. A computer electrocardiograph “VNS-Micro” (Neurosoft®, Ivanovo, Russia) was used for ECG recording. After 20 min of rest, participants were asked to stay supine quietly for 5.0 min for stationary condition HRV recording. Afterwards they were asked to stand up rapidly and remain in the standing position for 6 min – active orthostatic test (OT). RR intervals were determined with a sampling frequency of 2.0 kHz and were analyzed with “Poly-Spectrum” (Neurosoft®) software designed according to HRV standards. The time-domain parameters – standard deviation of normal RR intervals (SDNN), the square root of the mean squared differences of successive RR interval (RMSSD), and the percentage of differences between adjacent normal RR intervals exceeding 50 milliseconds (pNN50) were determined. The power spectral analysis was performed sequentially with a fast Fourier transformation. The following frequency-domain variables were studied: total power (TP, 0.01 to 0.40 Hz), high frequency power (HF, 0.15 to 0.40 Hz), low frequency power (LF, 0.04 to 0.15 Hz), and very low frequency power (VLF, 0.01 to 0.04 Hz). Occasional extrasystoles (not more than one per minute), as well as artifacts were detected *ad oculos* by an experienced physician (A.C.) and removed manually from the analysis. Extrasystoles were excluded from the analysis together with the consecutive compensatory pause, because they cause significant deviation of normal RR intervals with sinus rhythm and may distort the HRV evaluation.

### Clinical laboratory investigations, glycated hemoglobin, and ELISA

Routine clinical blood cell count was performed by automatic cell counter ABS-Micros 60-OT (Horiba Medical, Montpellier, France), and blood cell morphology evaluation was done by an experienced clinical laboratory specialist.

Fasting whole blood glucose was determined by the conventional glucose oxidase method routinely used in clinical laboratories, while glycated hemoglobin (HbA1c) was assessed using a highly sensitive method of ion-exchange liquid chromatography with a D-10TMSystem analyzer and BIO-RAD D-10TM reagents (Bio-Rad, Hercules, CA, USA). Commercially available ELISA-assay kits were used for determination of the levels of insulin, C-peptide, and aldosterone (DRG Instruments GmbH, Marburg, Germany), interleukin 6 (IL-6), interleukin 10 (IL-10), high sensitivity C-reactive protein (CRP) and tumor necrosis factor-α (TNF-α) (Vector-Best, Novosibirsk, Russia), thyroid stimulating hormone (TSH), cortisol, and testosterone (Chema, Moscow, Russia).

Surrogate methods for insulin resistance (IR)/sensitivity evaluation were also used. Homeostatic model assessment (HOMA) of IR is derived from the basal (fasting) levels of glucose and insulin is the most commonly used model, which highly correlates with the “gold standard” euglycemic clamp method (18), which is technically much more difficult to perform. HOMA-IR index was calculated using the formula HOMA-IR = fasting blood glucose × insulin/22.5.

### H.pylori determination

*H.pylori* antigen in stool was determined by immunochromatography with the use of monoclonal mice antibodies against *H.pylori*. Commercially available test kit “CITO TEST *H. pylori* Ag” (Pharmasco, Kyiv, Ukraine) was performed according to the manufacturer’s instructions. The manufacturer reports specificity as high as >99.0% and sensitivity >94.0%. The material for analysis was collected not earlier than two hours prior to testing, which was performed immediately after obtaining the sample in the laboratory.

### DHN-MA determination

Levels of the reduced and conjugated HNE metabolite, dihydroxynonane-mercapturic acid, were determined in aliquots (400 μL) of 2h-urine samples when available by competitive enzyme immunoassay using polyclonal antibodies and DHN-MA linked acetylcholinesterase as tracer, as described by Guéraud et al (11). Detection was performed at 414 nm using a plate reader (Thermo Labsystems, Cergy-Pontoise, France). Concentrations were calculated and expressed relative to the creatinine levels assayed in the same urine samples.

### Statistical analysis

All data were processed applying the statistical package Statistica 10.0 (Statsoft, Tulsa, OK, USA). Data were tested for normality of distribution using Shapiro-Wilk’s W-test. Data are presented as mean ± standard deviation (SD) or median (interquartile range). *t* test for independent samples or Mann-Whitney U-test were used. *P* value <0.05 was considered significant.

## Results

There were no significant differences between the groups in age, anthropometric and body composition parameters (Table 1).

**Table 1:** Age, anthropometric data, body composition, and hand dynamometry in *H. pylori* negative and positive asymptomatic sedentary participants, median (interquartile range)

Parameter	*H. pylori*-negative (n = 18)	*H. pylori*-positive (n = 12)	*P* (Mann-Whitney U-test)
Age (years)	30.0 (6.0)	30.0 (10.0)	1.000
Height (cm)	176.0 (9.0)	179.0 (5.0)	0.573
Body mass (kg)	77.6 (13.0)	79.25 (28.9)	0.983
Fat content (%)	22.8 (9.6)	20.65 (17.9)	0.917
Visceral fat (%)	7.0 (4.0)	6.50 (7.5)	0.983
Muscular mass (%)	37.4 (5.5)	38.65 (10.55)	0.983
Body mass index (kg/m^2^)	24.4 (3.7)	24.35 (7.85)	0.950
Dynamometry (right hand, kg)	49.5 (5.0)	48.5 (10.5)	0.632
Dynamometry (left hand, kg)	43.0 (9.0)	41.5 (9.0)	0.723

As it could be expected, most of HRV parameters in supine position were lower in *H.pylori* positive than in *H.pylori* negative participants (Table 2). More pronounced, but still not significant, differences were shown for parameters reflecting parasympathetic activity and autonomic balance, eg, pNN50 (*P* = 0.139), power of high frequency oscillations (*P* = 0.374), and LF/HF ratio (*P* = 0.175). However, the only significant difference was found for heart rate (*P* = 0.022), which was higher in *H.pylori* positive participants.

**Table 2:** Heart rate variability parameters in supine position in *H. pylori* negative and positive healthy sedentary participants, median (interquartile range)*

Parameter	*H. pylori*-negative (n = 18)	*H. pylori*-positive (n = 12)	*P* (Mann-Whitney U-test)
Heart rate, bpm	59.0 (8.0)	68.0 (9.0)	0.022
SDNN, ms*	51.5 (31.0)	48.5 (16.0)	0.458
RMSSD, ms*	49.0 (55.0)	36 (16.0)	0.212
pNN50, %*	30.1 (43.5)	11.89 (14.6)	0.139
Total power, ms^2^ × 10^3^	2.81 (3.07)	2.5 (1.82)	0.641
Very low frequency, ms^2^ × 10^3^	0.86 (1.03)	0.85 (0.94)	0.472
Low frequency, ms^2^ × 10^3^	0.86 (0.60)	1.05 (0.99)	0.499
High frequency, ms^2^ × 10^3^	0.80 (1.17)	0.56 (0.74)	0.374
Low frequency, normalized (%)	51.6 (35.7)	64.1 (32.9)	0.175
High frequency, normalized (%)	48.5 (35.7)	35.9 (32.9)	0.175
Low/high frequency ratio	1.08 (1.78)	1.79 (2.94)	0.175

*SDNN – standard deviation of normal RR intervals; RMSSD – the square root of mean squared differences of successive RR interval; pNN50 – the percentage of differences between adjacent normal RR intervals exceeding 50 milliseconds.

Similarly, in orthostatic test, parameters reflecting parasympathetic activity were higher in *H.pylori* negative participants (Table 3). In this group heart rate was lower (*P* = 0.068), pNN50 higher (*P* = 0.086) and ratio LF/HF significantly lower (*P* = 0.029) than in *H.pylori* positive participants.

**Table 3:** Heart rate variability parameters in orthostatic test in *H. pylori* negative and positive healthy sedentary participants, median (lower-upper quartile).

Parameter	*H. pylori*-negative (n = 18)	*H. pylori*-positive (n = 12)	*P* (Mann-Whitney U-test)
Heart rate, bpm	74.0 (7.0)	82.0 (17.0)	0.068
SDNN, ms*	54.5 (20.0)	47.5 (14.5)	0.309
RMSSD, ms*	26.0 (15.0)	19.5 (8.5)	0.117
pNN50, %*	5.93 (8.21)	1.84 (3.64)	0.086
Total power, ms^2^ × 10^3^	3.63 (2.41)	3.03 (2.57)	1.000
Very low frequency, ms^2^ × 10^3^	1.23 (1.22)	1.20 (0.56)	0.582
Low frequency, ms^2^ × 10^3^	1.26 (1.60)	1.27 (1.85)	0.446
High frequency, ms^2^ × 10^3^	0.25 (0.44)	0.21 (0.20)	0.236
Low frequency, normalized (%)	79.0 (15.4)	91.6 (17.0)	0.075
High frequency, normalized (%)	21.0 (15.4)	8.4 (17.0)	0.075
Low/high frequency ratio	3.81 (4.84)	10.90 (12.01)	0.029

*SDNN – standard deviation of normal RR intervals; RMSSD – the square root of mean squared differences of successive RR interval; pNN50 – the percentage of differences between adjacent normal RR intervals exceeding 50 milliseconds.

The homeostatic carbohydrate metabolism parameters, eg, fasting blood glucose and glycated hemoglobin were within normal range and no significant differences were observed between *H.pylori* negative and positive participants (*P* = 0.324 and *P* = 0.886 respectively, Figure 1A and B). These results indicate that the regulation of blood glucose level in *H.pylori* positive participants is not affected by the microorganism, or that initial functional changes are fully compensated. C-peptide levels were not significantly different between the groups (*P* = 0.606, Figure 1C), although a slight elevation in *H.pylori* positive participants was observed.

Fasting insulin levels were significantly higher in *H.pylori* positive participants (*P* = 0.037, Figure 2A). Consequently, HOMA-IR index was also significantly higher in the *H.pylori* positive group (*P* = 0.047, Figure 2B). This indicates that despite normal levels of glucose and glycated hemoglobin in *H.pylori* positive participants there are signs of insulin resistance. The maintenance of the glucose level in this group requires secretion of more insulin (Figure 1C), and fasting serum insulin level is higher (Figure 2B).

The levels of selected hormones, such as cortisol, thyroid stimulating hormone, testosterone, and aldosterone we determined in the study. However, no significant changes in the levels of these hormones were found (Figure 3A, B, C, and D). Moreover, the hormone levels of all participants were within the normal range (Table 4).

**Table 4:** Inflammatory markers in *H. pylori* negative and positive healthy sedentary participants, mean ± standard deviation

Parameter	*H. pylori*-negative (n = 18)	*H. pylori*-positive (n = 12)	*P* (*t* test)	Reference range
C-reactive protein (mg/mL)	4.42 ± 5.25	3.53 ± 3.01	0.598	0-8.0
Interleukin-6 (pg/mL)	3.07 ± 1.62	4.14 ± 1.63	0.089	0-10
Interleukin-10 (pg/mL)	9.55 ± 8.05	8.50 ± 5.01	0.691	0-20
Tumor necrosis factor-α (pg/mL)	3.66 ± 1.35	2.99 ± 1.80	0.167	0-6

The level of DHN-MA was determined in urine. In order to prevent the variations of its concentration related to the degree of natural urine dilution, we also determined the concentration of creatinine, so the ratio DHN-MA/creatinine adequately reflected the excretion of this metabolite. There was a slight elevation of DHN-MA adjusted by the level of creatinine in *H.pylori*-positive participants, but it did not reach the level of significance (*P* = 0.481) (Table 5).

**Table 5:** 1,4-Dihydroxynonane mercapturic acid (DHN-MA), creatinine, and their ratio in *H. pylori* negative and positive healthy sedentary participants, mean ± standard deviation

Parameter	*H. pylori*-negative (n = 18)	*H. pylori*-positive (n = 12)	*P* value (*t* test)
Dihydroxynonane-mercapturic acid (ng/mL)	6.02 ± 3.92	6.78 ± 5.66	0.690
Creatinine (mM/L)	14.39 ± 8.01	10.83 ± 5.50	0.159
Dihydroxynonane-mercapturic acid (ng/µmol creatinine)	0.48 ± 0.33	0.58 ± 0.39	0.481

## Discussion

The main findings of our paper include the following: *H.pylori* positive participants develop certain degree of insulin resistance and autonomic imbalance, however the levels of inflammatory biomarkers as well as DHN-MA (potential biomarker of oxidative stress) remain unaffected.

It was difficult to expect dramatic changes in HRV parameters in *H.pylori* positive asymptomatic sedentary participants since their general health condition was not significantly affected and they may be considered as apparently healthy. Therefore, detected mild changes in certain parameters seem to be encouraging for further studies. Parasympathetic activity is known to have anti-inflammatory properties, and various ways of vagal nerve output stimulation may reduce inflammatory responses in numerous animal models. The clinical importance of HRV decrease in terms of mechanisms and practical implications have been also recently discussed (19). These findings require further human studies in order to translate their results into medical practice (20), however, it is clear that the high activity of the parasympathetic nervous system provides numerous benefits for the organism and its suppression places the individual at risk.

This confirms the findings of other recent studies, indicating the relationships of insulin resistance and metabolic syndrome in *H.pylori* positive participants (2,5,6). Insulin resistance associated with *H.pylori* contamination along with sedentary lifestyle may be an important contributing factor of early development of cardiovascular disease, diabetes type 2, and metabolic syndrome. However, the exact mechanisms responsible for parasympathetic suppression and insulin resistance remain not fully understood. This issue is addressed by the second part of our study concerning the determination of oxidative stress marker – DHN-MA and inflammatory and hormonal profiles as possible contributing factors to the development of autonomic dysfunction and insulin resistance in *H.pylori* positive sedentary participants.

Since DHN-MA is a water soluble derivative of HNE excreted with urine, it was suggested to be a potential biomarker of oxidative stress available for noninvasive evaluation (18,21,22). In addition, the accumulation of HNE-histidine conjugates was shown in the gastric mucosa of patients with *H.pylori*-associated duodenal peptic ulcer (8). Therefore, in our study we used DHN-MA concentration in urine as an oxidative stress biomarker, and since we also hypothesized that insulin resistance in *H.pylori* positive participants may be caused by oxidative stress, we expected it to be elevated in *H.pylori* positive participants. However, we did not find any significant differences between the levels of DHN-MA and DHN-MA adjusted by the creatinine level in urine.

Thus, despite the evidence of autonomic dysfunction and insulin resistance development, neither oxidative stress index DHN-MA nor inflammatory markers were elevated in *H.pylori* positive asymptomatic sedentary participants. We suggest several possible explanations for these, in part unexpected, findings. First, metabolic and functional changes caused by *H.pylori* contamination in asymptomatic sedentary young men are mild compared to *H.pylori* associated diseases (chronic gastritis, peptic ulcer). Therefore, only some parameters have been affected. Second, considering the relatively low intensity of the microorganism’s impact in asymptomatic *H.pylori* carriers, an exposure time is not sufficient to exert any meaningful changes on the systemic level. And third, since DHN-MA was measured in urine, and the inflammatory markers in venous blood, it is likely, in our opinion, that the liver is able to detoxify the majority of waste products and toxins derived from the infectious process in the stomach, and therefore no changes were observed in systemic circulation and venous blood in particular.

The main limitation of the study was the sample size, which included 30 participants. However, strict inclusion/exclusion criteria together with careful selection of participants provided a nearly perfect distribution of study participants in two studied groups. Further studies may recruit more participants, as well as use new biomarkers for inflammation and oxidative stress evaluation. The strategies aimed to correct these early functional and metabolic changes in sedentary men may involve increased physical activity, intermittent fasting, or caloric restriction (23), and these changes should be extensively studied in the future not only in *H.pylori*-positive individuals, but also in other groups with insulin resistance.

## 

Figure 1. Fasting blood glucose (**A**), glycated hemoglobin (**B**) and C-peptide (**C**) in *H. pylori* negative and positive healthy sedentary participants (mean±SEM, *P*<0.05 was considered statistically significant; t-test).

Figure 2. Fasting serum insulin (**A**) and homeostatic model assessment – insulin resistance index (**B**) in *H. pylori* negative and positive healthy sedentary participants (mean±SEM, *P*<0.05 was considered statistically significant; t-test).

Figure 3. Levels of selected hormones in *H. pylori* negative and positive healthy sedentary participants (mean±SEM, *P*<0.05 was considered statistically significant; t-test).
